# Grafting Triggers Differential Responses between Scion and Rootstock

**DOI:** 10.1371/journal.pone.0124438

**Published:** 2015-04-13

**Authors:** Anita Kumari, Jitendra Kumar, Anil Kumar, Ashok Chaudhury, Sudhir P. Singh

**Affiliations:** 1 National Agri-Food Biotechnology Institute (NABI), Mohali, Punjab, India; 2 Guru Jambheshwar University of Science and Technology, Hisar, Haryana, India; University of North Carolina at Charlotte, UNITED STATES

## Abstract

Grafting is a well-established practice to facilitate asexual propagation in horticultural and agricultural crops. It has become a method for studying molecular aspects of root-to-shoot and/or shoot-to-root signaling events. The objective of this study was to investigate differences in gene expression between the organs of the scion and rootstock of a homograft (*Arabidopsis thaliana*). MapMan and Gene Ontology enrichment analysis revealed differentially expressed genes from numerous functional categories related to stress responses in the developing flower buds and leaves of scion and rootstock. Meta-analysis suggested induction of drought-type responses in flower buds and leaves of the scion. The flower buds of scion showed over-representation of the transcription factor genes, such as Homeobox, NAC, MYB, bHLH, B3, C3HC4, PLATZ etc. The scion leaves exhibited higher accumulation of the regulatory genes for flower development, such as SEPALLATA 1–4, Jumonji C and AHL16. Differential transcription of genes related to ethylene, gibberellic acid and other stimuli was observed between scion and rootstock. The study is useful in understanding the molecular basis of grafting and acclimation of scion on rootstock.

## Introduction

Grafting is a widely used and traditional method of asexual propagation in fruit crops which do not reproduce true-to-type from seed [1]. The benefits of grafting in vegetable crops are also being recognized in recent years [2]. Rootstocks influence the scion development in several ways, affecting the traits of agricultural interest, such as vegetative vigour, stress tolerance, yield, fruit quality etc. [2,3]. The controlling effect of rootstock over scion is possibly due to altered root-to-shoot and/or shoot-to-root chemical signaling [3]. Several studies on long-distance signaling via graft-union provide evidences for multiple types of mobile signals, such as hormones [4,5,6,7], proteins [8,9], ribonucleoprotein [10], RNAs [11], small RNAs [12,13,14,15,16], minerals [17,18] etc, conferring a wide range of effects on scion development.

Despite the wide use of grafting in agriculture, very little is known about the molecular mechanism of rootstock-regulation of scion’s phenotypes. Gene expression studies are useful approaches in understanding the genes involved in the effect of the rootstock. Transcriptional profiling in the scions of *Prunus cerasus* [19] and *Malus domestica* [20] revealed differences in the expression level of 99 and 116 transcripts, respectively, which could contribute to rootstock-regulation of biomass in scion. Recently, effect of heterografting has been examined on gene expression in scion [21] and graft interface [22] in *Vitis vinifera*. In the shoot apex of scion, grafted onto vigorous rootstock, the differentially expressed genes related to growth, stress, hormone signaling and hybrid vigour possibly confers vigour effects [21]. Up-regulation of stress response was notified at graft interface of heterografts, as compared to the homografts, suggesting that the tissues involved in graft-union could recognize and behaved differently in case of self or non-self grafting partner [22].

Grafting has become an experimental approach for studying plant biology, taking into account graft-transmissible long distance transport events and their impact on physiology of scion or rootstock, taking *Arabidopsis thaliana* as a model organism [23]. Since the first demonstration of inflorescence stem grafting in *A*. *thaliana* [24], several improvements have been made in the grafting protocol [23]. Recently, a modified wedge-style grafting of the primary inflorescence has been reported to obtain healthiest floral graft [25]. The aim of the study was to investigate the transcriptional profile in the organs of scion and rootstock in *A*. *thaliana*.

The present study analyses expression difference between scion and rootstock of a homograft in *A thaliana*. Microarray, a tool for accurate and high throughput gene expression analysis [26], was employed to examine transcriptome changes in the organs (flower bud and leaf) of scion and rootstock. The study furthers our understanding about the differential gene expression during flower and leaf development on scion and rootstock, and the genes involved in the acclimation of scion on rootstock after grafting.

## Material and Methods

### Plant material and growth conditions

The *Arabidopsis thaliana* var. Columbia-0 (Col-0) plants were used in the grafting experiments. The dried seeds were sterilized following the standard procedures, and were sown on Soilrite bed in pots. The pots were kept at 4°C in the dark for 2 days for stratification of seeds, and to synchronize seed germination. After stratification, the pots were shifted to PGC 20 growth chamber (Conviron, Canada) under long-day conditions (16-h light/8-h dark at 150 μmol m^-2^ s^-1^ irradiance) at 22°C ± 1°C and 65% humidity.

### Homografting

Homografting was carried out on young inflorescence stems of *A*. *thaliana* plants of uniform age (4–5 weeks) and height (~10 cm), following the procedure described by Nisar et al. [25], with some modifications. The primary inflorescence stem was cut horizontally by using a razor blade, and immediately placed in a petri dish containing sterile water; this part was used as scion for grafting on the same plant. A drop of water was placed on the cut end of the primary inflorescence stem of the same plant, to be used as rootstock. A vertical incision (~1 cm) was made in rootstock and the scion was cut in a wedge shape. Cut ends of the scion and rootstock were attached and wrapped with a parafilm around the graft. A support of a stick was provided to the plant. The plant was covered with a plastic bag to maintain high humidity for three days. The grafting was performed on multiple plants. Three grafted plants showing the best scion growth and development were selected for the study. The newly emerged un-opened flower buds and leaves were harvested from the side branches of scion and rootstock, at the same time. Harvesting of the samples was performed during 10 to 20 days after the graft (DAG). The samples were immediately frozen in liquid nitrogen and stored at -80°C till further use. The experiment was done in three independent biological replicates.

### RNA extraction and cDNA synthesis

Total RNA was extracted from the harvested leaf and flower bud samples using Spectrum Plant Total RNA kit (Sigma-Aldrich, USA), following the manufacturer’s instructions. On-column DNase (Sigma-Aldrich, USA) treatment was performed as instructed in the manual. The quality and concentration of total RNA were determined by using NanoQuant M200 Pro (Tecan) and agarose gel electrophoresis visualization. Double stranded cDNA synthesis, *in vitro* transcription to synthesize biotin labeled aRNA, purification and fragmentation of aRNA, and hybridization of arrays was performed following the protocol described in the technical manual of Affymetrix.

### Microarray

Affymetrix Arabidopsis ATH1 Genome Array GeneChip was used for microarray experiment. Affymetrix ATH1 GeneChip, a 3’ in vitro transcription (3’ IVT) expression array, contains more than 22,500 probe sets, representing approximately 24K genes. Labeling and hybridization of ATH1 GeneChips (one sample per chip) was performed according to the manufacturer’s instructions (http://www.affymetrix.com/support/technical/manuals.affx). The hybridized arrays were processed by running fluidics script FS450_0004 on an Affymetrix GeneChip Fluidics Station 450 and scanned on Affymetrix GeneChip Scanner 3000. The quality of hybridization was verified according to the Affymetrix microarray standards. The expression console of Affymetrix’s GeneChip Command Console (AGCC) software was used for computing cell intensity data of probesets and their positional values from image file. The intensities of probe arrays were normalized by using GeneSpring GX v12 (Agilent Technologies, Santa Clara, USA). The data has been submitted to NCBI (http://www.ncbi.nlm.nih.gov), with accession number GSE61631. Robust Multi-array normalization algorithm (RMA) values of probe sets were used for further statistical analysis. One-way ANOVA analysis was carried out in GeneSpring software with ‘Asymptotic’ *p* value computation and Benjamini-Hochberg false discovery rate (FDR) for multiple test correction (at *p* ≤ 0.05). The probe sets satisfying the criteria of p-value (≤ 0.05) and fold change (≥ 2) were used as differentially expressed genes for further analysis.

Functional annotation of the differentially expressed probe sets was obtained using the information available at TAIR (http://www.arabidopsis.org/tools/bulk/microarray/index.jsp) and PLEXdb (http://www.plexdb.org/modules/PD_general/tools.php). MapMan software was employed for visualization of differences in gene expression, and enrichment of functional categories in differentially expressed genes using the Wilcoxon rank-sum test (p value ≤ 0.05) [27,28].

Enrichment of Gene Ontology (GO) terms in the differentially expressed genes was performed using AgriGO analysis tool (http://bioinfo.cau.edu.cn/agriGO) [29], with Fisher tests and Bonferroni multiple testing correction (p ≤ 0.05). Kyoto Encyclopedia of Genes and Genomes (KEGG) categories was assigned by the plant gene set enrichment analysis toolkit (http://structuralbiology.cau.edu.cn/PlantGSEA/analysis.php) with fisher test function.

### Quantitative RT-PCR

For the validation of microarray data, quantitative RT-PCR was performed for five randomly selected genes in three biological replicates. cDNA was prepared from 500 ng of total RNA using Transcriptor First Strand cDNA Synthesis Kit (Roche, USA) according to manufacturer’s instructions. Gene expression was analyzed using 2X QuantiTect *SYBR Green* (Qiagen, USA), with a 200 nM primer concentration in a qRT-PCR machine (7500 Fast Real-Time PCR System, Applied Biosystems), according to the manufacturer’s instructions. The expression of genes of interest was normalized using housekeeping gene (polyubiquitin 10; At4g05320) and relative change in gene expression was quantified as described previously [30].

## Results and Discussion

### Homografting

**Fig 1:**
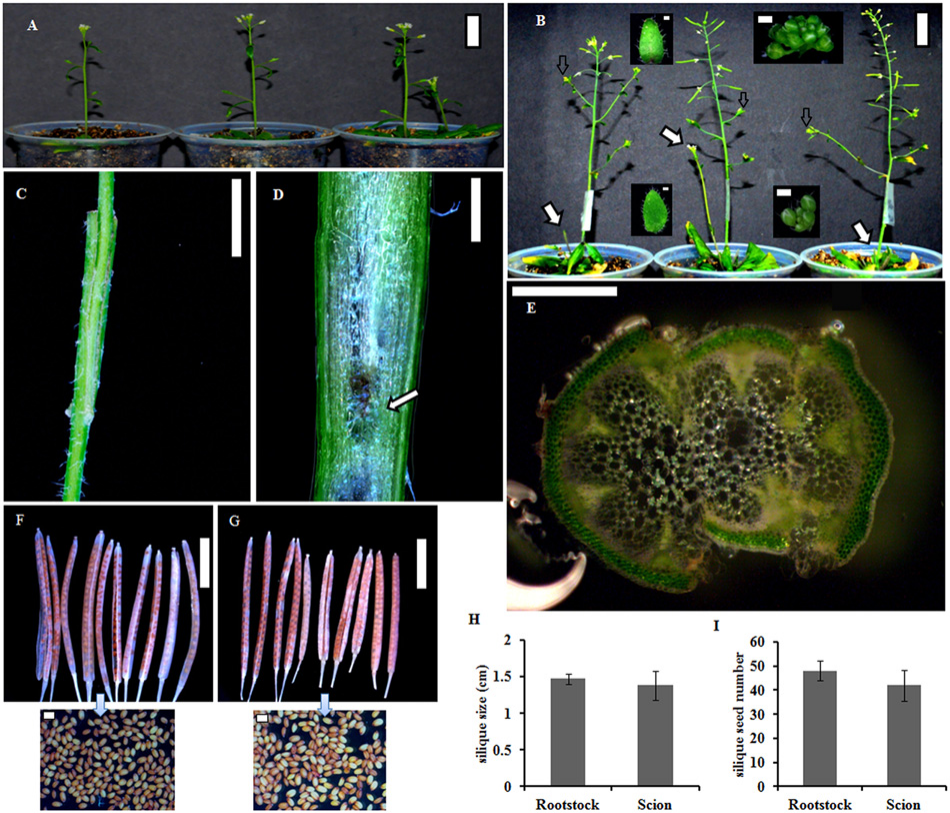
Homografting in *A*. *thaliana* plants. (A) Representative Arabidopsis plants selected for floral stem wedge-grafting (scale 2.5 cm). (B) Grafted plants (scale 2.5 cm) selected for harvesting the newly emerged un-opened flower buds and leaves (scale 500 μm) from the side branches of scion (up) and rootstock (down). The arrow shows rootstock in the plants. (C) A floral stem graft (10 DAG) showing wedge junction (scale 5 mm). (D) A longitudinal section through the floral stem graft (10 DAG) showing callus proliferation (arrow) near the wedge junction (scale 1 mm). (E) A transverse section from middle of the floral stem graft (10 DAG) (scale 500 μm). (F) Siliques (scale 1 mm) and seeds (scale 500 μm) of rootstock, and (G) scion. (H) Bar diagram representing length, and (I) seed number in mature siliques of rootstock and scion. The error bars indicate standard error in three biological replicates.

Homografting experiments were carried out on young primary inflorescence stems of Arabidopsis plants (Fig 1A). Though difficulties have been encountered in maintaining hydraulic turgor across the graft junction [23], with the recent developments in grafting technique [25], the integrity of the graft union formation has been improved. In a successful flowering stem graft, vascular connection is established by about 7 DAG [25]. The floral stem graft, which maintains shoot apical dominance with a taller primary stem, indicates a functional vascular connection at the graft junction [25]. Three floral stem grafts with the best scion growth and development (Fig 1B), indicating appropriate transport of water, nutrients and signalling molecules across the graft junction, were selected for the study. The longitudinal and transverse sections across the graft-junction (10 DAG) confirmed the establishment of vascular connections between scion and rootstock stems (Fig 1C–1E). Callus proliferation near the wedge junction (Fig 1D) is indicative of good regenerative growth, confirming the integrity of graft-union [25]. The newly emerged un-opened developing flower buds and leaves were harvested from the side branches of scion and rootstock (10–20 DAG), for gene expression analysis. At maturity, siliques of scion were comparable to that of rootstocks (Fig 1F–1I). However, the slight reduction in silique length and number of seeds could be due to grafting generated effects on scion development.

### Homografting alters the expression of many genes in flower buds and leaves

**Fig 2:**
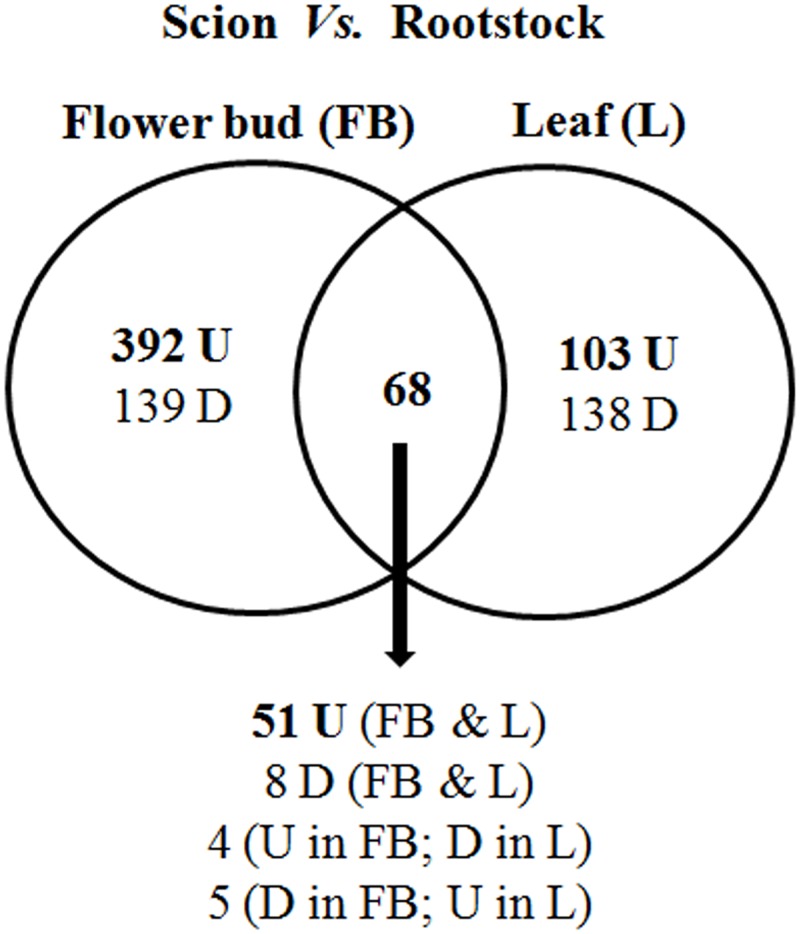
Venn diagram showing differentially expressed genes in flower bud and leaf (scion *vs*. rootstock; ≥ 2 fold change; p ≤ 0.05) (U = up-regulated, D = down-regulated). The details of the genes have been given in S1 Table.

The transcriptional changes were examined in flower buds and leaves of scion and rootstock, emerged during 10 to 20 days after the homograft. A total of 840 genes were identified as differentially expressed, by two folds or more at p ≤ 0.05, in flower buds and/or leaves of scion and rootstock (Fig 2, S1 Table). The fold expression of five randomly selected genes was validated by qPCR analysis (S1 Fig). The differentially expressed genes have been further analyzed and discussed.

### Divergent profiles of differentially expressed genes in flower buds and leaves

MapMan, AgriGO, and KEGG categorization of the differentially expressed genes revealed a differential level of accumulation of a divergent set of genes in flower buds and leaves of scion as compared to rootstock (Tables 1–6). Up-regulated hormonal metabolism was observed in flower buds of both scion and rootstock. However, several genes related to hormonal signaling pathways were over-represented in scion buds, conferring tolerance to stress [31]. Higher accumulation of the transcripts for Late Embryogenesis Abundant (LEA) proteins in flower buds of scion could protect cellular proteins from aggregation, under the abiotic stresses such as desiccation, osmotic stresses, temperature, salinity etc [32]. The transcription factors, such as homeobox genes, MADS box and MYB which express in responses to several stresses [33,34,35,36,37], and hormonal stimuli [33,38,39], were up-regulated in flower buds of the scion. In addition, a few genes involved in protein synthesis and photosynthesis were up-regulated in the flower buds of scion, as compared to that of rootstock (Table 1). In contrast to scion, genes associated with the MapMan functional categories of cellular processes, cell organization, CHO metabolism, and F-box proteins which are critical for the controlled degradation of cellular proteins, were up-regulated in the flower buds of rootstock (Table 1). This could be indicative of comparatively increased rate of cell division in flower buds of rootstock.

**Table 1:** MapMan functional categories (BINs) for significantly up-regulated (2 ≥ fold) genes in the flower buds of scion and rootstock.

Bin number	Bin name	Elements	p-value
**Up-regulated in scion**			
33.2	Development late embryogenesis abundant	5	2.48E-03
27.3.22	RNA.regulation of transcription.HB,Homeobox transcription factor family	7	3.03E-03
17	Hormone metabolism	25	2.60E-02
17.1	Hormone metabolism.abscisic acid	7	4.98E-02
17.1.3	Hormone metabolism.abscisic acid.induced-regulated-responsive-activated	2	2.59E-02
17.5	Hormone metabolism.ethylene	7	1.49E-02
17.5.1	Hormone metabolism.ethylene.synthesis-degradation	4	4.32E-03
29.2	protein.synthesis	5	2.95E-02
27.3.26	RNA.regulation of transcription.MYB-related transcription factor family	2	2.98E-02
27.3.24	RNA.regulation of transcription.MADS box transcription factor family	2	4.29E-02
1.1.1	PS.lightreaction.photosystem II	6	4.53E-02
1.1.1.2	PS.light reaction.photosystem II.PSII polypeptide subunits	6	4.53E-02
**Up-regulated in rootstock**			
31	Cell	9	8.00E-04
31.1	Cell organisation	5	1.60E-03
17.5	Hormone metabolism.ethylene	5	2.74E-02
17.5.2	Hormone metabolism.ethylene.signal transduction	4	1.94E-02
3	Minor CHO metabolism	2	3.56E-02
3.4	Minor CHO metabolism.myo-inositol	2	3.56E-02
3.4.4	Minor CHO metabolism.myo-inositol.myo inositol oxygenases	2	3.56E-02
29.5.11.4.3.2	Protein.degradation.ubiquitin.E3.SCF.FBOX	3	4.47E-02

The details of the genes are given in S4 Table.

**Table 2:** MapMan functional categories (BINs) for significantly up-regulated (2 ≥ fold) genes in the leaves of scion and rootstock.

Bin number	Bin name	Elements	p-value
**Up-regulated in scion**			
34	Transport	9	2.44E-02
34.99	Transport.misc	2	2.60E-02
11.6	Lipid metabolism. Lipid transfer proteins	3	3.79E-02
**Up-regulated in rootstock**			
26	Misc	17	1.66E-03
26.16	Misc.myrosinases-lectin-jacalin	3	2.72E-02
29	protein	7	3.28E-02
27.3.26	RNA.regulation of transcription.MYB-related transcription factor family	2	4.74E-02
20.1.7.12	Stress.biotic.PR-proteins.plant defensins	2	4.92E-02

The details of the genes are given in S4 Table.

**Table 3:** AgriGO categories for significantly up-regulated (2 ≥ fold) genes in the flower buds of scion and rootstock.

GO term	Ontology	Description	Contingency	p-value
**Up-regulated in scion**				
GO:0010876	P	Lipid localization	11, 15, 435, 22464	7.9E-13
GO:0009414	P	Response to water deprivation	18, 155, 428, 22324	8.6E-09
GO:0009415	P	Response to water	18, 64, 428, 22315	1.9E-08
GO:0009719	P	Response to endogenous stimulus	39, 748, 407, 21731	1E-07
GO:0009725	P	Response to hormone stimulus	37, 687, 409, 21792	1E-07
GO:0042221	P	Response to chemical stimulus	65, 1684, 381, 20795	3.4E-07
GO:0009737	P	Response to abscisic acid stimulus	20, 255, 426, 22224	5.1E-07
GO:0051179	P	Localization	61, 1621, 385, 20858	1.8E-06
GO:0019915	P	Lipid storage	6, 15, 440, 22464	2.2E-06
GO:0019953	P	Sexual reproduction	9, 56, 437, 22423	4.5E-06
GO:0010033	P	Response to organic substance	41, 974, 405, 21505	8.9E-06
GO:0009738	P	Abscisic acid mediated signaling pathway	9, 63, 437, 22416	1.1E-05
GO:0015833	P	Peptide transport	9, 63, 437, 22416	1.1E-05
GO:0006857	P	Oligopeptide transport	9, 63, 437, 22417	1.1E-05
GO:0050896	P	Response to stimulus	94, 3107, 352, 19372	2.3E-05
GO:0009788	P	Negative regulation of abscisic acid mediated signaling pathway	5, 14, 441, 22465	2.5E-05
GO:0006810	P	Transport	55, 1556, 391, 20923	3.4E-05
GO:0051234	P	Establishment of localization	55, 1562, 391, 20917	3.8E-05
GO:0015979	P	Photosynthesis	12, 145, 434, 22334	6.1E-05
GO:0022900	P	Electron transport chain	9, 84, 437, 22395	8.4E-05
GO:0009628	P	Response to abiotic stimulus	41, 1083, 405, 21396	0.00009
GO:0009968	P	Negative regulation of signal transduction	6, 34, 440, 22445	0.00011
GO:0010648	P	Negative regulation of cell communication	6, 34, 440, 22446	0.00011
GO:0055114	P	Oxidation reduction	13, 187, 433, 22292	0.00016
GO:0009624	P	Response to nematode	7, 54, 439, 22425	0.00018
GO:0006091	P	Generation of precursor metabolites and energy	16, 278, 430, 22201	0.00023
GO:0009755	P	Hormone-mediated signaling pathway	14, 223,432, 22256	0.00024
GO:0032870	P	Cellular response to hormone stimulus	14, 223,432, 22257	0.00024
GO:0016491	F	Oxidoreductase activity	54, 1302, 392, 21177	4.7E-07
GO:0005215	F	Transporter activity	48, 1128, 398, 21351	1.1E-06
GO:0022857	F	Transmembrane transporter activity	37, 853, 409, 21626	1.3E-05
GO:0022891	F	Substrate-specific transmembrane transporter activity	30, 677, 416, 21802	6.1E-05
GO:0022804	F	Active transmembrane transporter activity	25, 521, 421, 21958	7.7E-05
GO:0015144	F	Carbohydrate transmembrane transporter activity	10, 105, 436, 22374	8.5E-05
GO:0022892	F	Substrate-specific transporter activity	33, 794, 413, 21685	8.6E-05
GO:0015295	F	Solute:hydrogen symporter activity	9, 86, 437, 22393	9.9E-05
GO:0005402	F	Cation:sugar symporter activity	9, 86, 437, 22394	9.9E-05
GO:0005351	F	Sugar:hydrogen symporter activity	9, 86, 437, 22395	9.9E-05
GO:0008324	F	Cation transmembrane transporter activity	20, 375, 426, 22104	0.00011
GO:0015294	F	Solute:cation symporter activity	10, 111, 436, 22368	0.00013
GO:0015293	F	Symporter activity	10,112, 436, 22367	0.00014
GO:0009055	F	Electron carrier activity	22, 455, 424, 22024	0.00019
GO:0051119	F	Sugar transmembrane transporter activity	9, 95, 437, 22384	0.0002
GO:0016021	C	Integral to membrane	32, 510, 414, 21969	3.1E-08
GO:0031224	C	Intrinsic to membrane	39, 858, 407, 21621	2.7E-06
GO:0005576	C	Extracellular region	20, 378, 426, 22101	0.00012
GO:0009523	C	Photosystem II	6, 39, 440, 22440	0.00022
**Up-regulated in rootstock**				
GO:0007018	P	Microtubule-based movement	5, 51, 147, 22428	3.70E-05
GO:0003777	F	Microtubule motor activity	5, 66, 147, 22413	0.00012
GO:0003774	F	Motor activity	5, 87, 147, 22392	0.00039

The details of the genes are given in S4 Table.

**Table 4:** AgriGO categories for significantly up-regulated (2 ≥ fold) genes in the leaves of scion and rootstock.

GO term	Ontology	Description	Contingency	p-value
**Up-regulated in scion**				
GO:0010876	P	Lipid localization	10, 15, 149, 22734	6.60E-16
GO:0010584	P	Pollen exine formation	8, 14, 151, 22735	1.50E-12
GO:0010927	P	Cellular component assembly involved in Morphogenesis	8, 18, 151, 22461	7.00E-12
GO:0010208	P	Pollen wall assembly	8, 18, 151, 22462	7.00E-12
GO:0048646	P	Anatomical structure formation involved in Morphogenesis	10, 85, 149, 22394	1.30E-09
GO:0006869	P	Lipid transport	10, 113, 149, 22366	1.70E-08
GO:0009555	P	Pollen development	9, 110, 150, 22369	1.70E-07
GO:0012505	C	Endomembrane system	43, 2768, 116, 19711	4.70E-07
GO:0022607	P	Cellular component assembly	11, 221, 148, 22258	7.90E-07
GO:0048229	P	Gametophyte development	9, 163, 150, 22316	3.70E-06
GO:0048869	P	Cellular developmental process	13, 386, 146, 22093	5.10E-06
GO:0008289	F	Lipid binding	10, 227, 149, 22252	7.10E-06
GO:0048856	P	Anatomical structure development	23, 1232, 136, 21247	2.30E-05
GO:0032989	P	Cellular component morphogenesis	9, 221, 150, 22258	3.70E-05
GO:0045229	P	External encapsulating structure organization	8, 179, 151, 22300	5.50E-05
GO:0009791	P	Post-embryonic development	13, 501, 146, 21978	7.20E-05
GO:0032501	P	Multicellular organismal process	24, 1479, 135, 21000	0.00013
GO:0009653	P	Anatomical structure morphogenesis	12, 463, 147, 22016	0.00014
GO:0033036	P	Macromolecule localization	11, 404, 148, 22075	0.00018
GO:0007275	P	Multicellular organismal development	23, 1426, 136, 21053	0.0002
GO:0032502	P	Developmental process	25, 1644, 134, 20835	0.00026
GO:0044085	P	Cellular component biogenesis	11, 431, 148, 22048	0.0003
GO:0044464	C	Cell part	111, 12783, 48, 9696	0.00056
GO:0005623	C	Cell	111, 12783, 48, 9697	0.00056
GO:0016747	F	Transferase activity, transferring acyl groups other than amino-acyl groups	7, 221, 152, 22258	0.0012
GO:0030528	F	Transcription regulator activity	23, 1628, 136, 20851	0.0012
GO:0008415	F	Acyltransferase activity	6, 165, 153, 22314	0.0013
GO:0003700	F	Transcription factor activity	21, 1448, 138, 21031	0.0015
**Up-regulated in rootstock**				
GO:0050896	P	Response to stimulus	51, 3107, 99, 19372	4.00E-10
GO:0051707	P	Response to other organism	19, 465, 131, 22014	5.50E-10
GO:0009607	P	Response to biotic stimulus	19, 507, 131, 21972	2.20E-09
GO:0051704	P	Multi-organism process	20, 603, 130, 21876	6.00E-09
GO:0006952	P	Defense response	19, 622, 131, 21857	5.20E-08
GO:0010033	P	Response to organic substance	23, 974, 127, 21505	1.80E-07
GO:0045087	P	Innate immune response	11, 265, 139, 22214	2.40E-06
GO:0006955	P	Immune response	11, 280, 139, 22199	4.00E-06
GO:0002376	P	Immune system process	11, 280, 139, 22199	4.00E-06
GO:0006950	P	Response to stress	28, 1766, 122, 20713	1.70E-05
GO:0042221	P	Response to chemical stimulus	27, 1684, 123, 20795	2.10E-05
GO:0009723	P	Response to ethylene stimulus	7, 134, 143, 22354	4.40E-05
GO:0009814	P	Defense response, incompatible interaction	6, 97, 144, 22382	6.50E-05
GO:0010200	P	Response to chitin	6, 115, 144, 22364	0.00016
GO:0009719	P	Response to endogenous stimulus	15, 748, 135, 21731	0.00017
GO:0009611	P	Response to wounding	6, 132, 144, 22347	0.00032
GO:0009753	P	Response to jasmonic acid stimulus	6, 146, 144, 22333	0.00054
GO:0030246	F	Carbohydrate binding	7, 139, 143, 22340	5.50E-05
GO:0012505	C	Endomembrane system	44, 2768, 106, 19711	2.70E-08
GO:0005618	C	Cell wall	13, 514, 137, 21965	5.10E-05
GO:0030312	C	External encapsulating structure	13, 518, 137, 21961	5.50E-05

The details of the genes are given in S4 Table.

**Table 5:** KEGG categories for significantly up-regulated (2 ≥ fold) genes in the flower buds of scion and rootstock.

Description	Hits	Total	p-value
**Up-regulated in scion**			
Tropane, piperidine and pyridine alkaloid biosynthesis	3	18	4.53E-03
Cysteine and methionine metabolism	5	64	5.25E-03
Stilbenoid, diarylheptanoid and gingerol biosynthesis	5	66	5.93E-03
Limonene and pinene degradation	5	69	7.05E-03
Tyrosine metabolism	3	24	9.27E-03
Phenylalanine metabolism	5	80	0.0124
Methane metabolism	5	82	0.0137
Glyoxylate and dicarboxylate metabolism	3	30	0.0161
Biosynthesis of alkaloids derived from ornithine, lysine and nicotinic acid	7	166	0.0231
Fatty acid elongation in mitochondria	1	2	0.0478
**Up-regulated in rootstock**			
Ascorbate and aldarate metabolism	2	31	0.0137
Inositol phosphate metabolism	2	37	0.0188

The details of the genes are given in S4 Table.

**Table 6:** KEGG categories for significantly up-regulated (2 ≥ fold) genes in the scion leaf.

Description	Hits	Total	p-value
**Up-regulated in scion**			
Flavonoid biosynthesis	2	19	6.39E-03
Biosynthesis of phenylpropanoids	5	247	0.0153
Phenylpropanoid biosynthesis	3	104	0.0238
**Up-regulated in rootstock**			
Alpha-Linolenic acid metabolism	3	28	6.54E-04
Metabolism of xenobiotics by cytochrome P450	2	21	7.04E-03
Alanine, aspartate and glutamate metabolism	2	41	0.0234
Glutathione metabolism	2	52	0.0356
Plant-pathogen interaction	3	138	0.043

The details of the genes are given in S4 Table.

Enrichment of Gene Ontology (AgriGO) terms in the differentially expressed genes revealed that most of the genes up-regulated in the flower buds of scion, belonged to the biological process of responses to different stimuli (chemical, abiotic stresses and hormone etc.), localization, transport, and oxidoreductase activities (Table 3). The GO terms related to microtubule-based movement and motor activities, involved in cytoskeleton organization and developmental processes [33], were enriched in flower buds of rootstock (Table 3). The leaves of scion showed a comparatively high level of accumulation of genes associated with transport and lipid metabolism (Tables 2 and 4). Rootstock leaves accumulated the transcripts predicted to be involved in responses to stress, biotic and abiotic stimuli, and defense and wound responses (Tables 2 and 4).

The functional significance of genes was also explored by KEGG, which exhibited significant over-representation of genes associated with amino acid metabolism, and biosynthesis of other secondary metabolites such as alkaloids, stilbenoid, diarylheptanoid, and gingerol in flower buds (Table 5), and flavonoid and phenylpropanoid biosynthesis in leaf (Table 6) of scion. Alkaloids have several biological significance of being active stimulators, inhibitors and terminators of growth [40]. The genes could participate in several regulation mechanisms and confer protection against environmental stresses to the plant organs [41]. In rootstock, the genes associated with ascorbate, aldarate and inositol phosphate metabolism were up-regulated in flower buds (Table 5), whereas leaves exhibited active amino acid metabolism, glutathione and alpha-linolenic acid metabolism, and plant-pathogen interaction (Table 6).

### Meta-analysis

Similarity search meta-analysis was performed against 3287 diverse collections of Arabidopsis microarray data sets listed in Genevestigator, by using the differentially expressed transcripts between scion and rootstock. The perturbation showing maximum similarity with our data comprised the transcriptional comparison between drought study *vs* control plants in flower buds and leaves (S2 Fig). The meta-analysis suggests that grafting induces drought-type responses in flower buds and leaves of scion, which could play a role in acclimation to grafting induced stresses.

### Differential expression of transcription factors in flower buds and leaves

**Fig 3:**
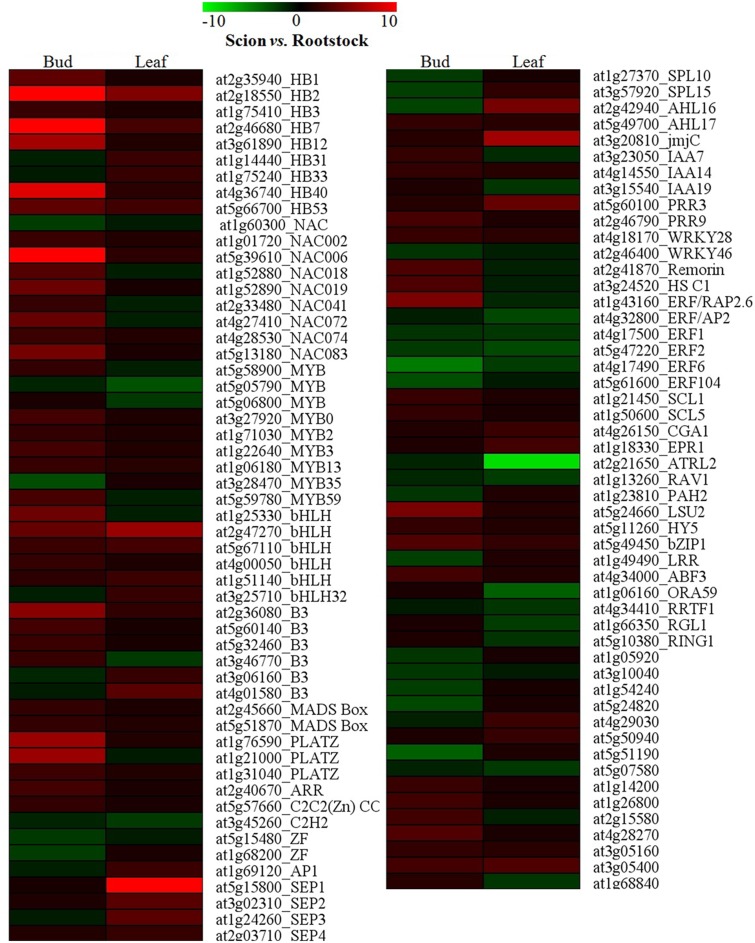
A heatmap of differentially expressed transcripts related to transcription factors in flower bud and leaf (scion *vs*. rootstock; ≥ 2 fold change; p ≤ 0.05). The color scale at the top of each heat map shows expression values in fold change. The details of the genes have been mentioned in S2 Table.

Out of about 25,500 genes, around 2000 transcription factor genes have been recognized in Arabidopsis genome [42]. Transcriptional regulation plays a pivotal role in temporal and spatial control over gene expression in plants. Altered expression levels of transcription factors were observed in flower buds and leaves developed on scion and rootstock (Fig 3).

Homeobox transcription factor family (HB) genes were significantly up-regulated in flower buds (e.g. at2g18550, at2g46680, at4g36740, at5g66700 and at3g61890) and leaves of scion (at2g18550, at2g46680, at5g66700, at1g14440, at1g75240), as compared to rootstock. In several studies, the expression regulation of homeobox genes by different abiotic and biotic stimuli has been inferred [33,34,38,43]. The higher level of transcript accumulation of homeobox genes in the vegetative and reproductive organs of scion could be helpful in achieving tolerance to subsequent stresses after grafting.

MYB transcription factor super family play regulatory roles in differentiation, metabolism and development processes, and defense responses in plants [35]. Scion flower buds showed higher expression of MYB genes which are known to regulate and/or respond during cell cycle (at5g59780) [44], differentiation (trichome initiation) (at3g27920) [46], phenylpropanoide pathway (at1g22640) [46], abiotic stress responses (e.g. drought, light and wounding) (at1g06180) [45]and abiotic and biotic stimuli (at1g71030 and at5g58900) [47]. However, the expression of MYB35/TDF1 (at3g28470), essential for anther tapetum development [48], was observed down regulated by three folds in scion flower buds. This could be indicative of grafting effect on tapetum development. Surprisingly, up-regulation of the MYB genes was not observed in scion leaves at a significant level. On the other hand, rootstock leaves exhibited enhanced expression of the MYB-related genes having role in signal transduction (at5g06800) [49], and express in response to abiotic or biotic stimuli (at5g05790) [47].

Basic helix-loop-helix (bHLH) gene family members (about 160 in Arabidopsis) are universal transcription factors in eukaryotes; however, the biological roles of the bHLH genes are poorly understood in plants. Some of the bHLH TFs, up-regulated in scion flower buds, are known to participate in regulating biosynthesis of the sterol derivatives-brassinosteroids (at1g25330) [50], transcription of peroxidases to balance reactive oxygen species (ROS) (at2g47270) [51], early gynoecium development (at5g67110) [52], and double fertilization (at4g00050) [53]. Scion leaves showed enhanced level of expression of bHLH TFs which regulate stomata movement and photoperiodism (at1g51140) [53,54]and anthocyanin biosynthesis (at3g25710) [53].

The transcripts for B3-type TFs (at2g36080, at5g60140, at5g32460 and at3g46770), involved in flower development [55], and drought responsive PLATZ family TFs (at1g76590, at1g21000 and at1g31040) [53,56], were abundant in scion flower buds. Expression of stress-inducible NAC transcription factors (at5g39610, at5g13180, at1g52890, at1g01720, at4g28530 and at2g33480) was up-regulated at significant levels in scion flower buds. The NAC transcription factors have previously been shown to be ABA, drought and NaCl-inducible [57].

The ethylene response factor, AP2- RAP2.6 (at1g43160), was significantly up-regulated in scion flower buds. It functions in plant response to various abiotic and biotic stresses, possibly through ABA-dependent pathway [58,59]. The importance of RAP2.6 has been emphasized in achieving water-stress tolerance in plant tissues [59]. This was further corroborated by visualization of abundance of genes associated with phytohormones, mainly ABA, that could play important roles in mediating responses to various stresses in scion flower buds (Fig 3). Enhanced transcript accumulation of key regulators of sulfur assimilation pathway, LSU2 (at5g24660) [60]and HY5 (at5g11260) [61], could be indicative of implications of stress, presumably water stress [60], on the sulfur assimilation in scion flower buds. Drought conditions affect the regulation of sulfur assimilation in plant tissues, and it has been anticipated as a fertile ground for new discoveries connecting primary sulfur metabolism with the stress responses, mainly drought [62]. The expression elevation of bZIP1 (at5g49450), a positive regulator of plant tolerance to salt, osmotic and drought stresses [63], water-deficit stress related remorin family protein (at2g41870) [64]and the stress responsive heat shock C1 (at3g24520) [53]anticipates role of these genes in acclimating scion on rootstock.

**Fig 4:**
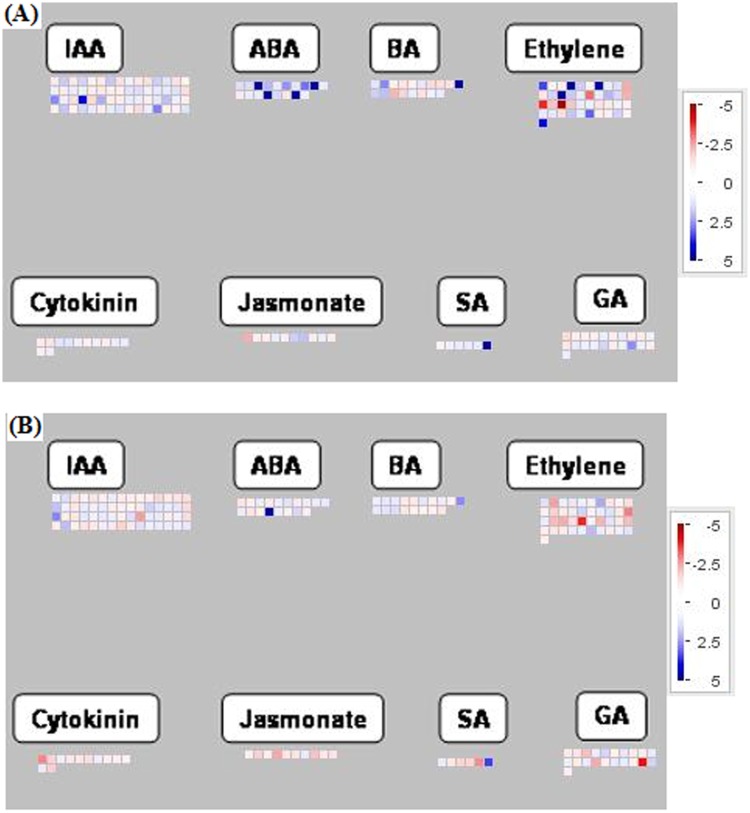
MapMan visualization of differentially expressed genes (scion *vs*. rootstock; p ≤ 0.05) assigned to the functional category of hormone metabolism in (A) flower bud, and (B) leaf. The details of the genes have been mentioned in S3 table.

The E genes of ABCDE model, SEPALLATA (SEP) 1–4, are essential for the normal development of petals, stamens, carpels and sepals [65]. SEP 1–4 genes (at5g15800, at3g02310, at1g24260 and at2g03710) were up-regulated in the leaves of scion, as compared to rootstock. Mutation in SEP genes leads to development of flowers composed of leaf-like organs, whereas, over-expression promotes early flowering without affecting floral morphology [66]. In addition, some other key regulators of flowering, Jumonji C and AHL16, were up-regulated in scion leaves. Jumonji C (at3g20810) is a histone demethylase which regulates the period length in Arabidopsis by chromatin remodeling [67]. AHL16 is an AT-hook DNA binding protein, which regulates vegetative to the reproductive phase transition of the meristem, and flowering time [68]. The enhanced level of transcripts for SEP 1–4, AHL16 and Jumonji C genes could be suggestive of the initiatives taken by scion for regulating flowering. The MYB transcription factor, ATRL2 (at2g21650), was highly expressed in rootstock leaves. The gene has been observed to be involved in stress responses [53,69], besides in ovule development and control of floral asymmetry [70]. Rootstock leaves showed elevated transcription of genes related to ethylene (at4g17500, at5g47220, at4g17490, at4g32800, at5g07580 and at1g13260) and gibberellic acid (at1g66350 and at5g56300). Thus, enhanced expression was observed for the genes related to ethylene and gibberellic acid in scion flower buds and rootstock leaves (Fig 4).

### Highly expressed genes

A total of 18 genes (other than transcription factors) were identified with an expression difference (scion *vs* rootstock) of at least 8 folds in flower buds and leaves (Table 7). The transcript level of dehydration and ABA inducible genes- ABA responsive protein-related (at3g02480) [56], Histone H1-3 (at2g18050) [71], Responsive to ABA 18 dehydrin family protein (at5g66400) [71]and protein phosphatase 2C (at1g07430 and at3g11410) [72,73], were higher by many folds in scion flower buds and leaves (Table 7). This indicates existence of ABA-mediated signaling in scion which could help the scion organs in withstanding grafting related stress, such as water deficiency. This is further supported by higher level of expression of Late Embryogenesis Abundant-7 protein in scion buds which confers resistance to abiotic stresses and ABA sensitivity [74]. The grafting may induce ethylene production in scion flower buds, as suggested by the several fold up-regulation of 1-Aminocyclopropane-1-carboxylate (ACC) oxidase which is involved in the final step of ethylene production in plant tissues [75,76]. Expression of Cysteine Endopeptidase 1 was also found highly expressed, which could be related to ethylene regulation [77]or in response to stress stimuli [78]in scion flower buds. Another ethylene induced and flowering related gene, Xyloglucan Endotransglucosylase/hydrolase3 [79], was up-regulated in scion flower buds. In scion buds, no gene was down-regulated with a difference of at least 8 folds.

**Table 7:** Genes (other than TFs) showing at least 8-fold (bold) differential expression (scion *vs* rootstock) in flower buds and/or leaves.

Putative gene function	GeneID	Fold Change in flower bud	Fold change in leaf
Histone H1-3	at2g18050	**27.04**	**8.24**
Responsive to ABA18	at5g66400	**20.01**	1.59
2-Oxoglutarate-dependent dioxygenase	at2g25450	**18.92**	2.34
ABA-responsive protein-related	at3g02480	**18.69**	**8.70**
Cysteine proteinase	at5g50260	**17.29**	1.26
1-Aminocyclopropane-1-carboxylate (ACC) oxidase	at1g12010	**16.33**	-1.19
Late embryogenesis abundant protein	at1g52690	**13.59**	7.87
Protein phosphatase2C	at1g07430	**11.47**	1.82
Xyloglucan endotransglycosylase/hydrolase3	at3g25050	**11.15**	-1.00
Response to cyclopentenone	at2g31945	**9.34**	5.48
Cell wall / vacuolar inhibitor of fructosidase1	at1g47960	**9.29**	1.96
Cinnamyl-alcohol dehydrogenase (CAD) family	at1g09500	**8.33**	**9.38**
Rapid alkalinization factor (RALF) family protein	at4g14020	**8.03**	1.48
Cold Regulated Gene27	at5g42900	-1.06	**9.91**
Defensin-like (DEFL) family protein	at3g59930	1.69	**9.88**
Cinnamyl-alcohol dehydrogenase (CAD) family	at1g09500	**8.33**	**9.38**
Lipid transfer protein4	at5g59310	3.25	**9.10**
Glucosinolate biosynthetic process	at3g45160	1.04	**-9.20**

The details of the genes have been given in S4 Table.

Scion leaves showed higher accumulation of the transcripts for Histone H1-3, ABA-responsive protein-related, Cold Regulated Gene27 (at5g42900) [80], Defensin-like protein (at3g59930) [81], Cinnamyl-alcohol dehydrogenase (at1g09500) [81]and Lipid transfer protein4 (at5g59310) [81]. In rootstock leaves, the transcript for a gene of Glucosinolate biosynthetic process (at3g45160) was highly up-regulated. Glucosinolate biosynthesis is known to be involved in defense signaling pathways, and its expression is induced in response to salicylic acid, jasmonic acid, ethylene and wound [82]. It coincides with up-regulation of the transcripts related to salicylic acid, jasmonic acid, and ethylene in the rootstock leaves (Fig 4).

## Conclusion

Grafting triggers differential expression of numerous genes related to stress, biotic and abiotic stimuli, hormonal pathway, and flowering etc. in flower buds and leaves of the scion and rootstock. The study is useful in understanding the molecular basis of grafting and the intermediates involved in the acclimation of scion on rootstock.

## Supporting Information

S1 Fig Quantitative RT-PCR expression analyses of five randomly chosen genes. The relative expression of the five genes was in agreement with the microarray fold change. The sequences of primers and details were provided in S5 Table.
(TIF)

S2 Fig Meta analysis. The similarity search in Genevestigator, using the differentially expressed transcripts (scion *vs*. rootstock; ≥ 2 fold change; p ≤ 0.05) revealed perturbations (top 3) comparing transcriptome between the drought study *vs*. control plants in flower buds and leaves.
(TIF)

S1 Table Differentially regulated probe sets with ≥ 2 fold change expression difference at p ≤ 0.05, between scion *vs*. rootstock in flower bud and/or leaf. (XLSX)

S2 Table Details of the differentially expressed genes showed in Fig 3. (XLSX)

S3 Table Details of the differentially expressed genes showed in Fig 4. (XLSX)

S4 Table Details of the differentially expressed genes mentioned in Tables 1–6. (XLSX)

S5 Table Gene-specific primers used for qRT-PCR. (XLSX)
