# Supplementary figures and images for: Grafting Triggers Differential Responses between Scion and Rootstock
Source: PLoS One. 2015 Apr 13;10(4):e0124438. doi: 10.1371/journal.pone.0124438 (PMC4395316; doi:10.1371/journal.pone.0124438)

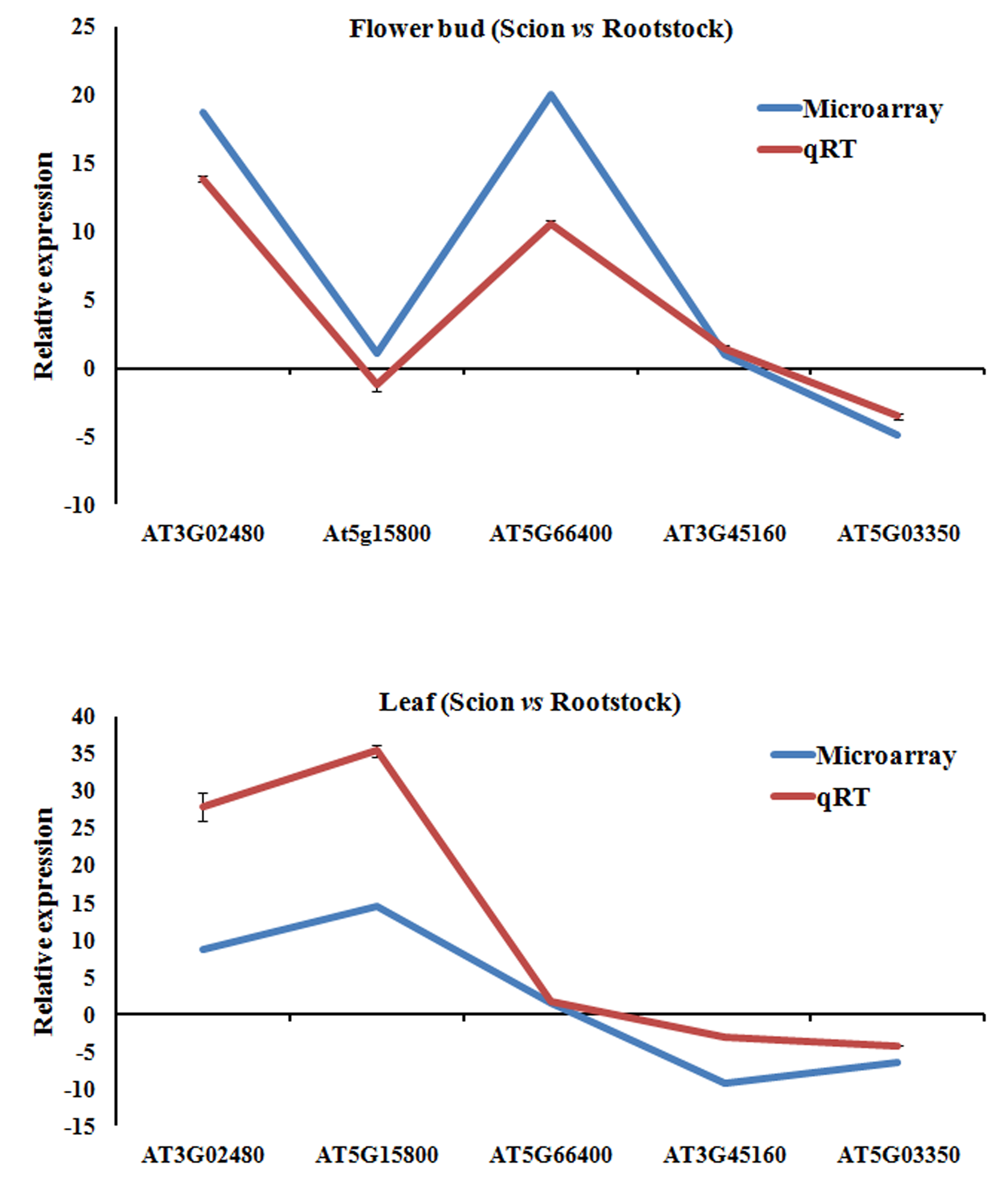

Supplement: S1 Fig — The relative expression of the five genes was in agreement with the microarray fold change. The sequences of primers and details were provided in S5 Table. (TIF) [file pone.0124438.s001.tif]

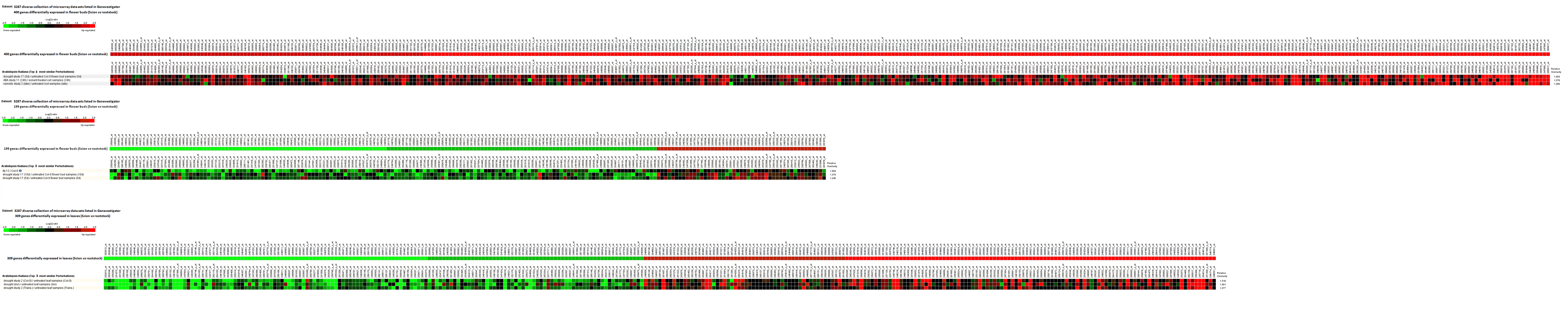

Supplement: S2 Fig — The similarity search in Genevestigator, using the differentially expressed transcripts (scion vs. rootstock; ≥ 2 fold change; p ≤ 0.05) revealed perturbations (top 3) comparing transcriptome between the drought study vs. control plants in flower buds and leaves. (TIF) [file pone.0124438.s002.tif]
